# Supplementary figures and images for: Selection of Ethanol Tolerant Strains of Candida albicans by Repeated Ethanol Exposure Results in Strains with Reduced Susceptibility to Fluconazole
Source: bioRxiv. 2023 Nov 10:2023.09.13.557677. Originally published 2023 Sep 14. Preprint. [Version 2] doi: 10.1101/2023.09.13.557677 (PMC10515905; doi:10.1101/2023.09.13.557677)

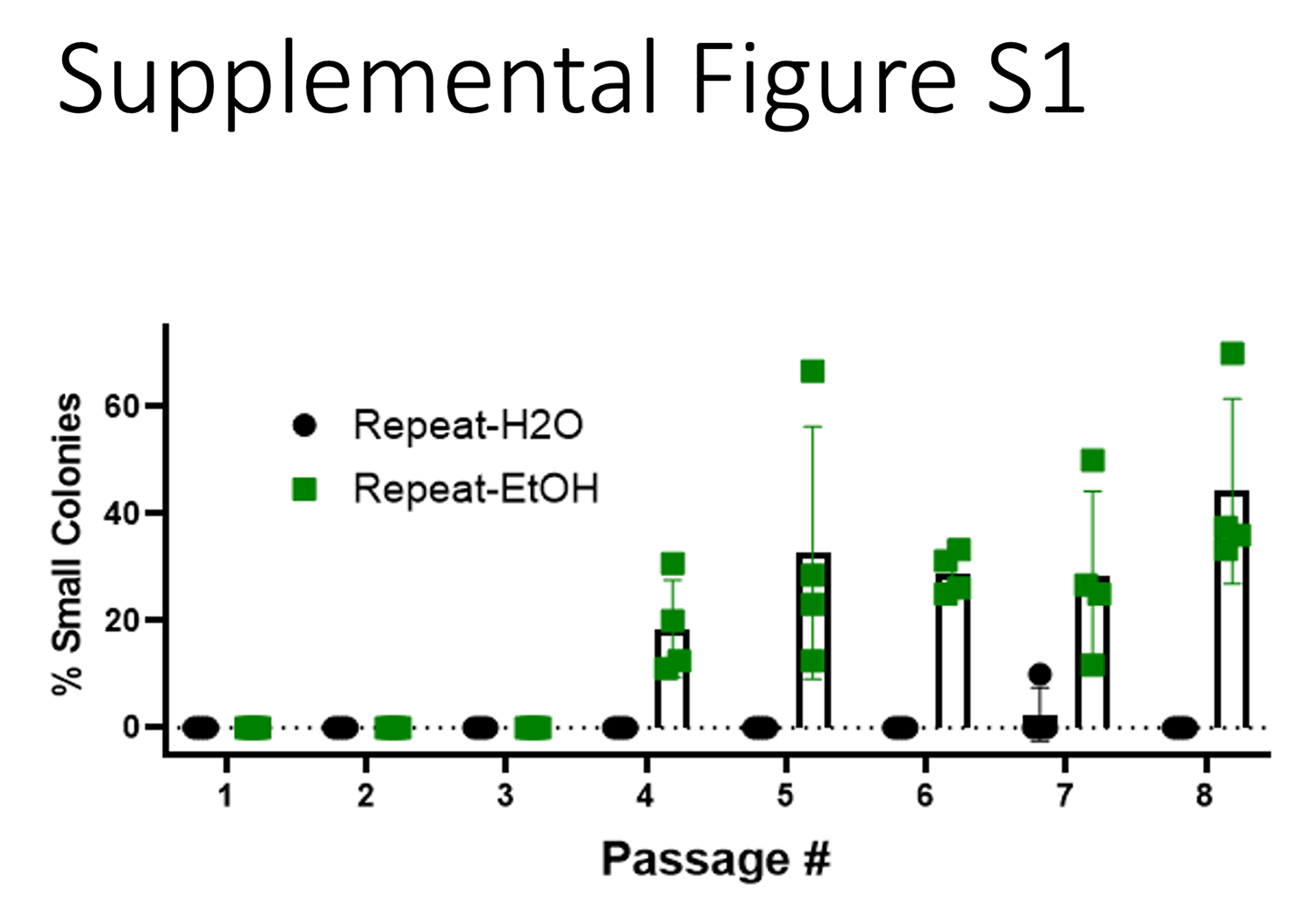

Supplement: Supplement 1 — Supplemental Figure S1: Quantification of small colony variants throughout passaging of four separate populations of cells that were exposed repeatedly to increasing concentrations of ethanol in medium A or repeatedly exposed to water in medium A. Quantification of small colony variants throughout passage numbers of four separate populations of cells that were exposed repeatedly to increasing concentrations of ethanol in media A or repeatedly exposed to water in media A. Green squares were repeatedly exposed to ethanol and black dots were repeatedly exposed to water. Y-axis shows % small colonies relative to total number of colonies. Bars represent the mean values of the populations at that passage number. Small colonies were classified as less than 50% of the size of the normal sized colonies on a plate by eye. [file media-1.tif]

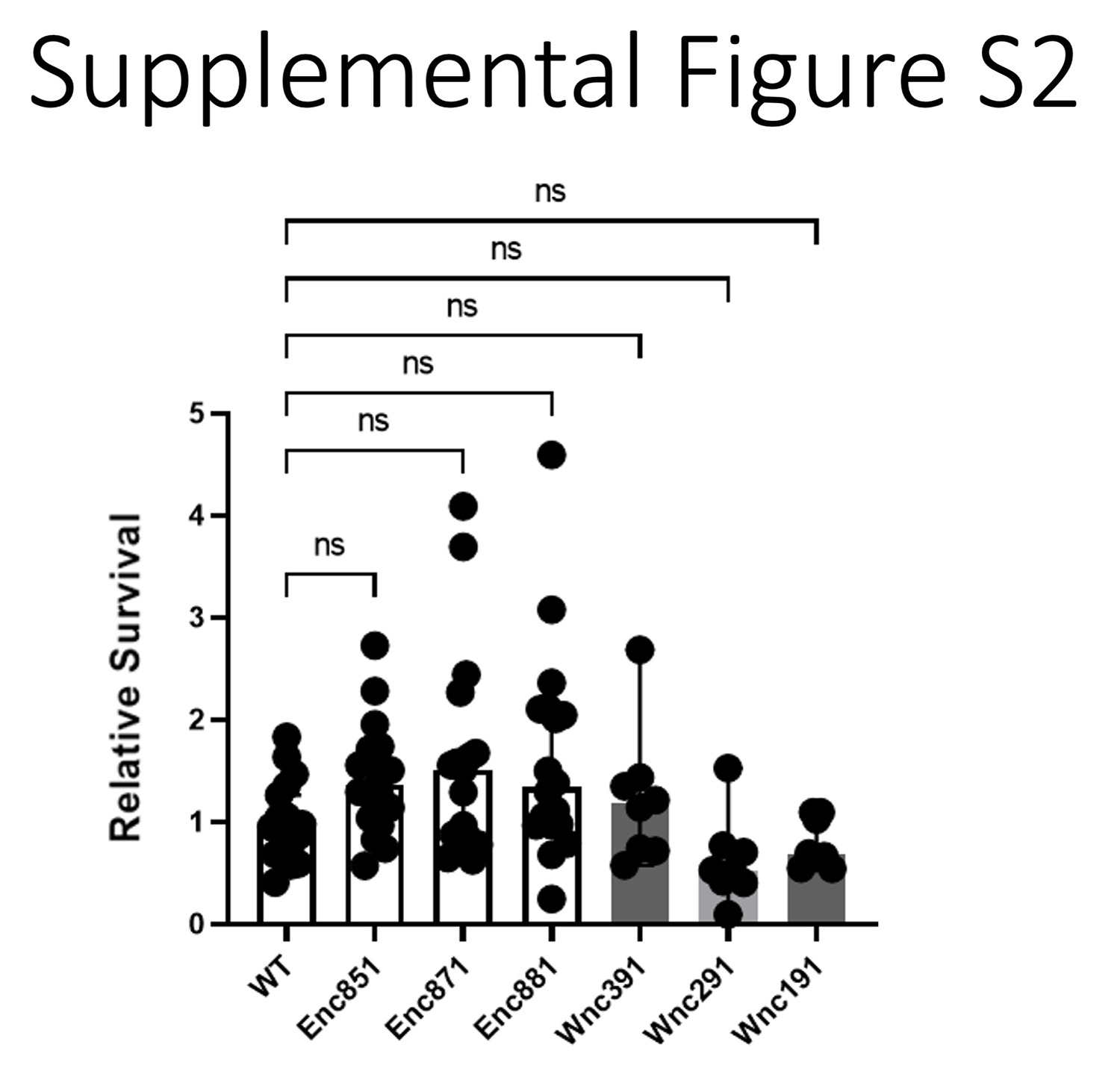

Supplement: Supplement 2 — Supplemental Figure S2: Enc and Wnc survival in 10% ethanol. Enc and Wnc survival in 10% ethanol. Colonies exposed for 4 hours in 10% ethanol or 10% water and plated on YPD-agar to count final colonies. The percentage of final colonies in 10% ethanol compared to 10% water were calculated and used to calculate a relative survival compared to SC5314 within each experiment (y-axis). Normal-sized colonies from ethanol populations (Enc) or water population (Wnc) were used. One-way ANOVA with Welch’s correction was used for statistical testing with multiple comparisons. Median values with 95% confidence interval are plotted for 8–16 replicate cultures from 2–4 separate experiments for each strain. [file media-2.tif]

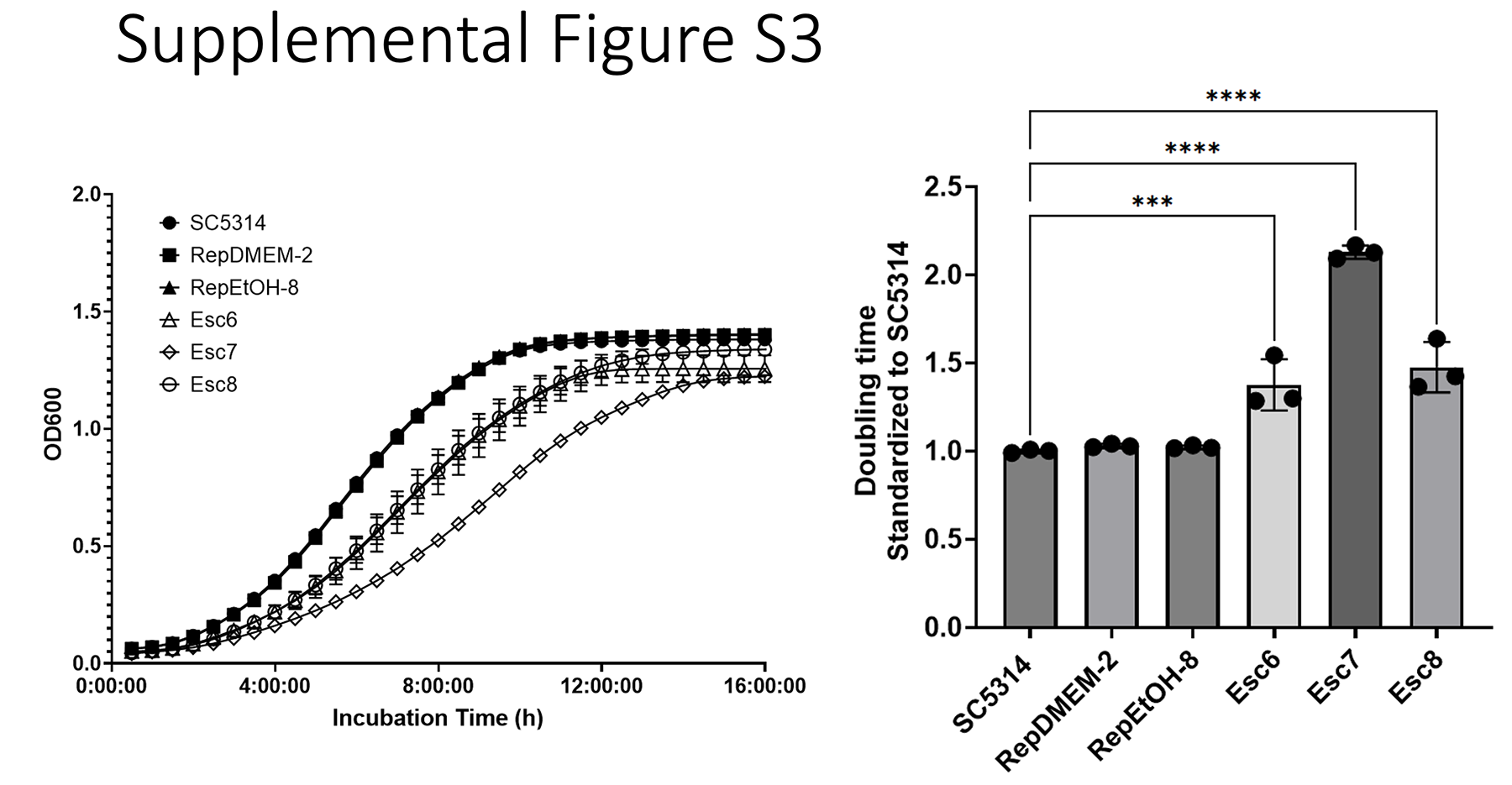

Supplement: Supplement 3 — Supplemental Figure S3: Growth curves and doubling times of Esc strains and normal-sized colony strains in YPD. Isolated colonies were picked and grown for 20 hours to post-exponential phase and seeded into YPD in 96 well plates at an approximate OD600 of 0.05. Cells were grown for 48 hours at 30°C with constant shaking in a Biotek Epoch2 plate reader with OD600 read every 30 minutes. Three biological replicate cultures from each strain were used to graph the growth curves. Growth curves show the mean and standard deviation of each timepoint. The wildtype, background strain: SC5314, a culture from the repeat media-A (with no ethanol exposure) is shown as RepDMEM-2, and a normal sized colony from a repeat ethanol exposure population is shown as RepEtOH-8. Esc6, Esc7, and Esc8 are all shown as well. Timepoints 0–24 hours were used to calculate doubling times by performing a nonlinear regression in GraphPad Prism with the exponential plateau fit to find the rate constant: k. All R2 values were above 0.9331. k values were then used to calculate doubling times with the formula LN(2)/k. These were standardized to the SC5314 doubling times and then plotted in this figure as standardized doubling times. [file media-3.tif]

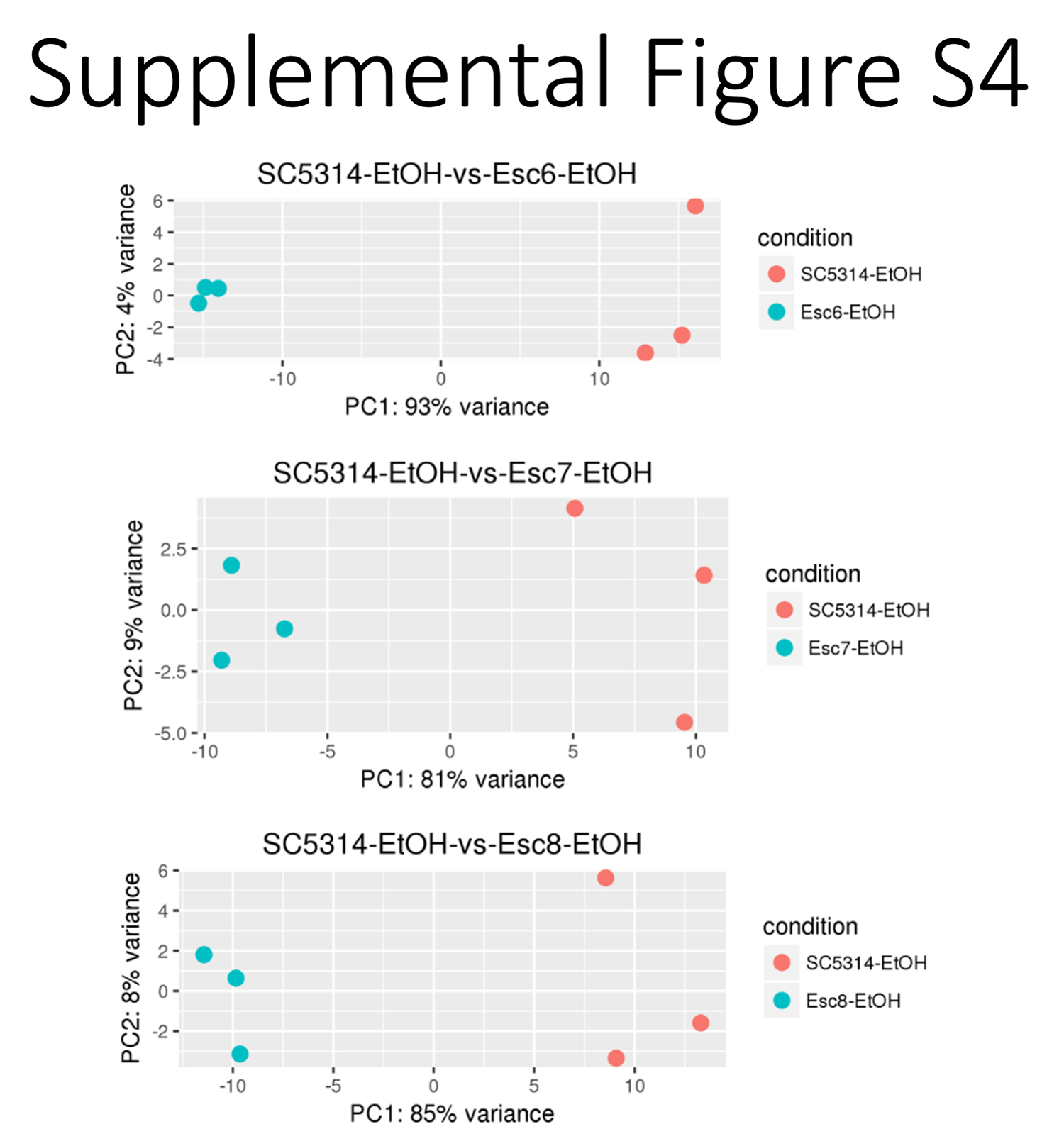

Supplement: Supplement 4 — Supplemental Figure S4: Principal component analyses for SC5314 vs Esc6, Esc7, or Esc8 in 10% ethanol from RNA sequencing. Principal component analyses for SC5314 vs Esc6, Esc7, or Esc8 in 10% ethanol each from RNA sequencing. PC1 variance ranges from 81%–93% and PC2 variance ranges from 4%–9%. SC5314 is shown in red dots in each and Esc strains are shown in blue. Each dot represents a separate biological replicate culture that was sequenced. [file media-4.tif]

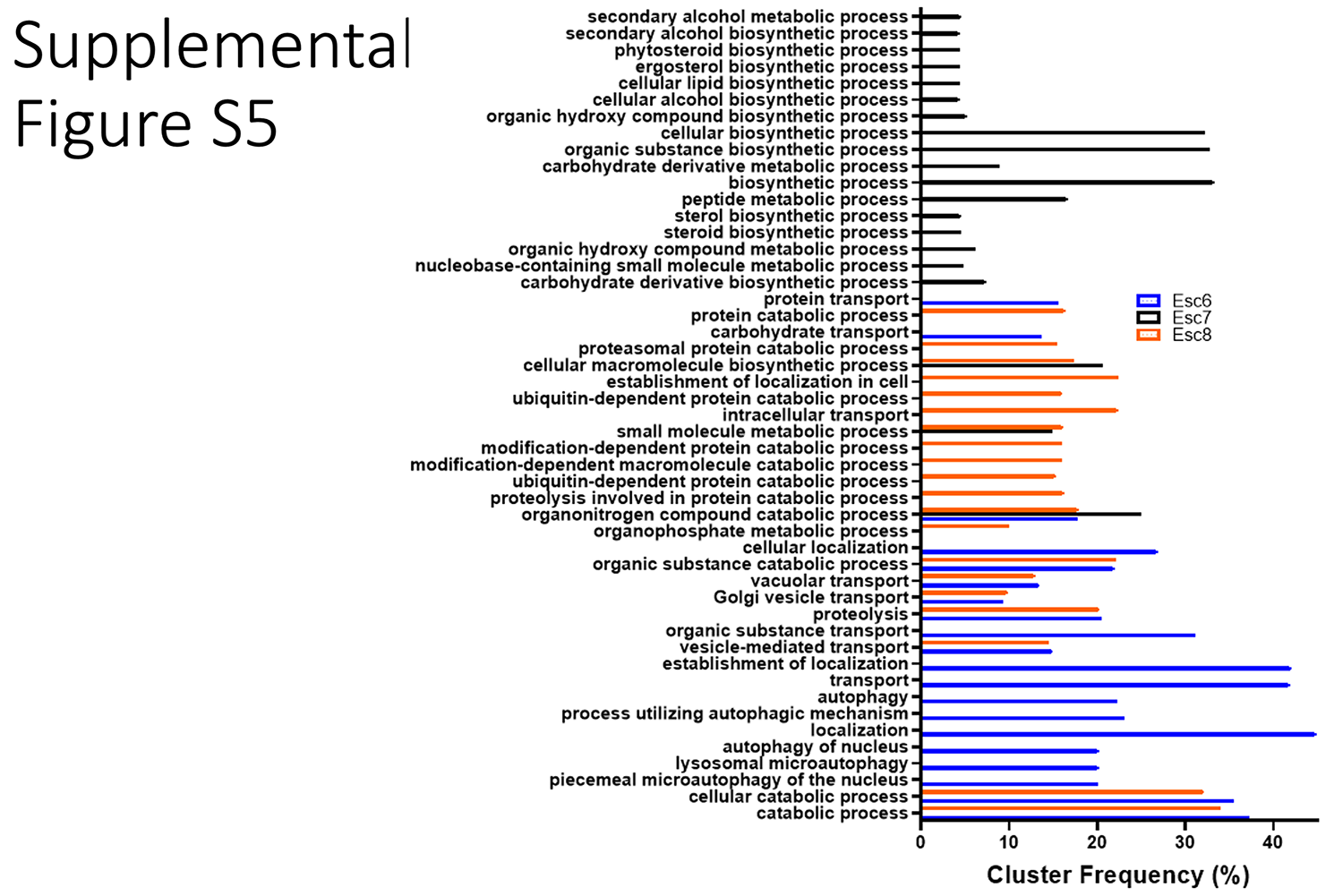

Supplement: Supplement 5 — Supplemental Figure S5: Gene Ontology Terms of the Top 20 Processes of Significantly Enriched Transcripts in Esc strains exposed to ethanol compared to SC5314 exposed to ethanol. Gene Ontology Terms of the Top 20 Processes of Significantly Enriched Transcripts in Esc strains exposed to ethanol compared to SC5314 exposed to ethanol. Transcripts that were significantly enriched in Esc strains were used to determine GO term process clustering on Candida Genome Database GO term finder. The top 20 hits from each strain were added to the graph and shown on y-axis. Esc6 bars are shown in blue, Esc7 bars are shown in black, and Esc8 bars are shown in orange. X-axis shows the cluster frequency of each of these processes relative to the total number of genes in the respective sets of enriched transcripts used for the analysis. [file media-5.tif]

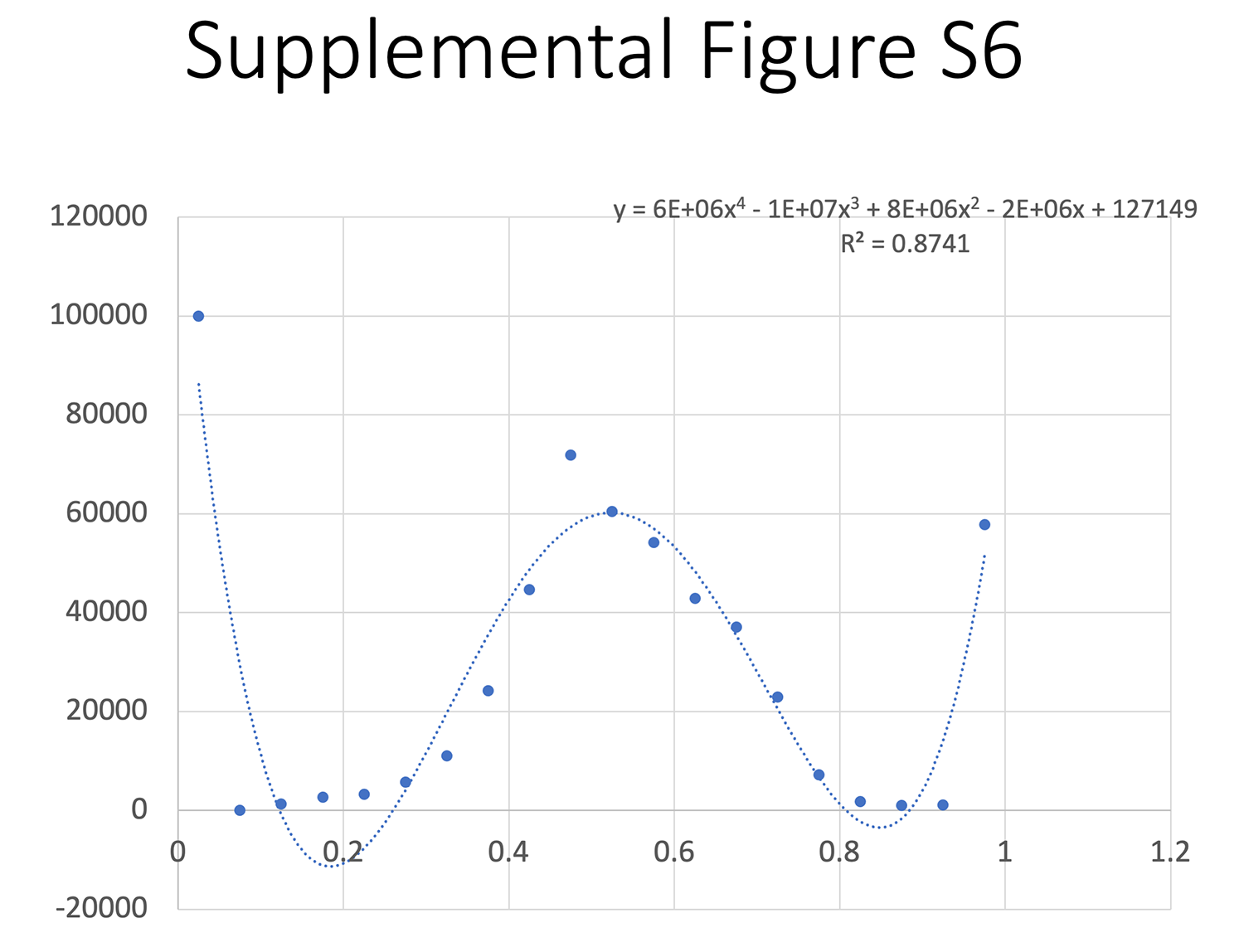

Supplement: Supplement 6 — Supplemental Figure S6: Histogram Fit Curve of Esc6 Allele Depth Ratio. Histogram fit curve of Esc6 allele depth ratio is shown. Esc6 is used as an example of what was done for all of the strains that were sequenced. Binning was performed by calculating the number of genes that had allele depth ratios within bins of 0.05. The bin of 0–0.05 was set to an arbitrary number as this represents homozygous for the reference strain and should not be called in the sequence variant report. Then a trimodal fit was performed in excel and minima values were calculated and used for further determination of heterozygous and homozygous polymorphisms. [file media-6.tif]

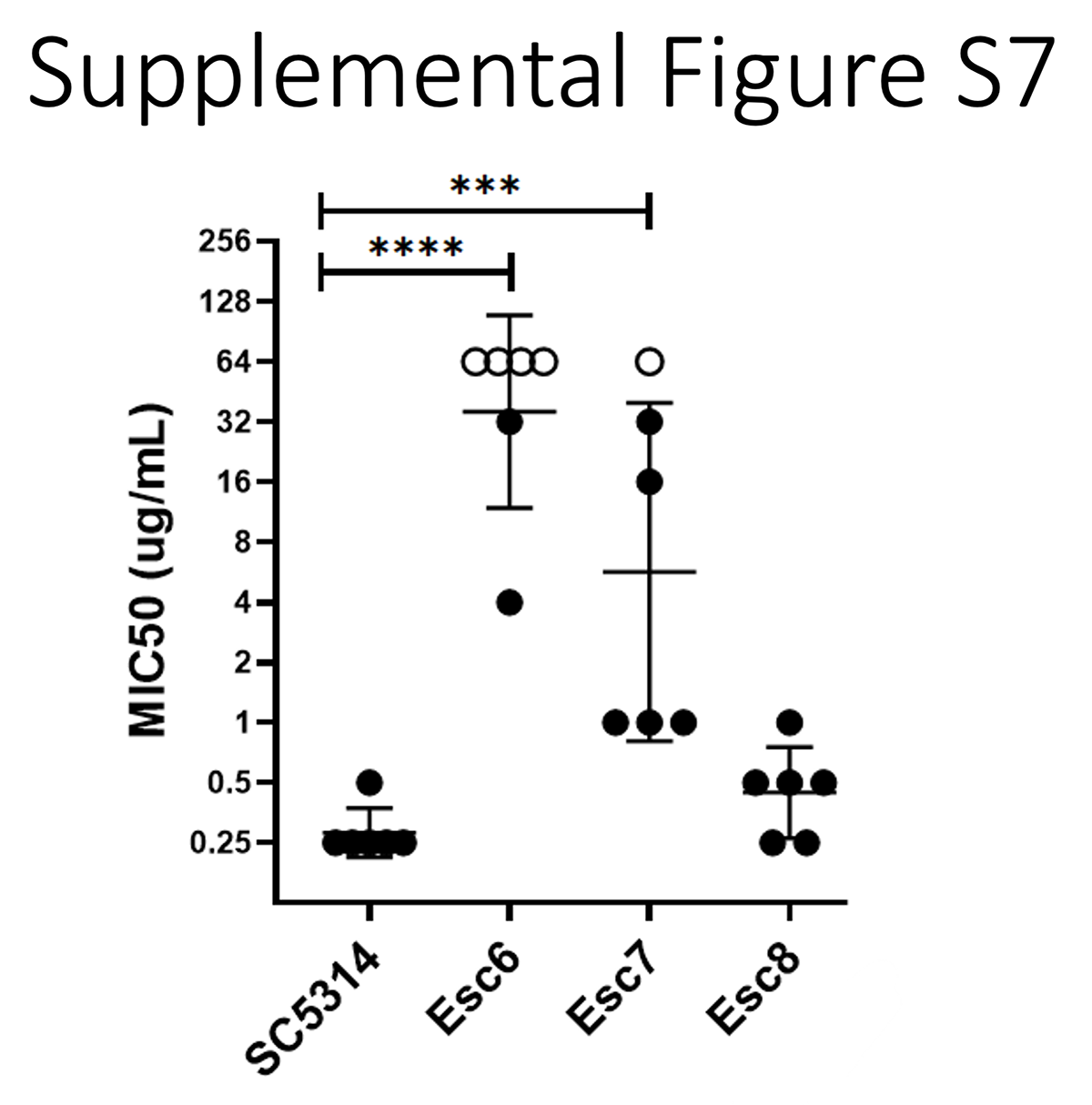

Supplement: Supplement 7 — Supplemental Figure S7: MIC50 values from broth-microdilution experiments of SC5314 and Esc6, Esc7, and Esc8. MIC50 values from broth-microdilution experiments of SC5314 and Esc6, Esc7, and Esc8. Six separate biological replicate experiments were conducted and plotted. MIC50 values were determined for each by measuring metabolic activity (XTT) and determining the well that represented a 50% reduction in metabolic activity. Geometric means with geometric SD are graphed and ordinary One-way ANOVA was performed for statistical significance. [file media-7.tif]

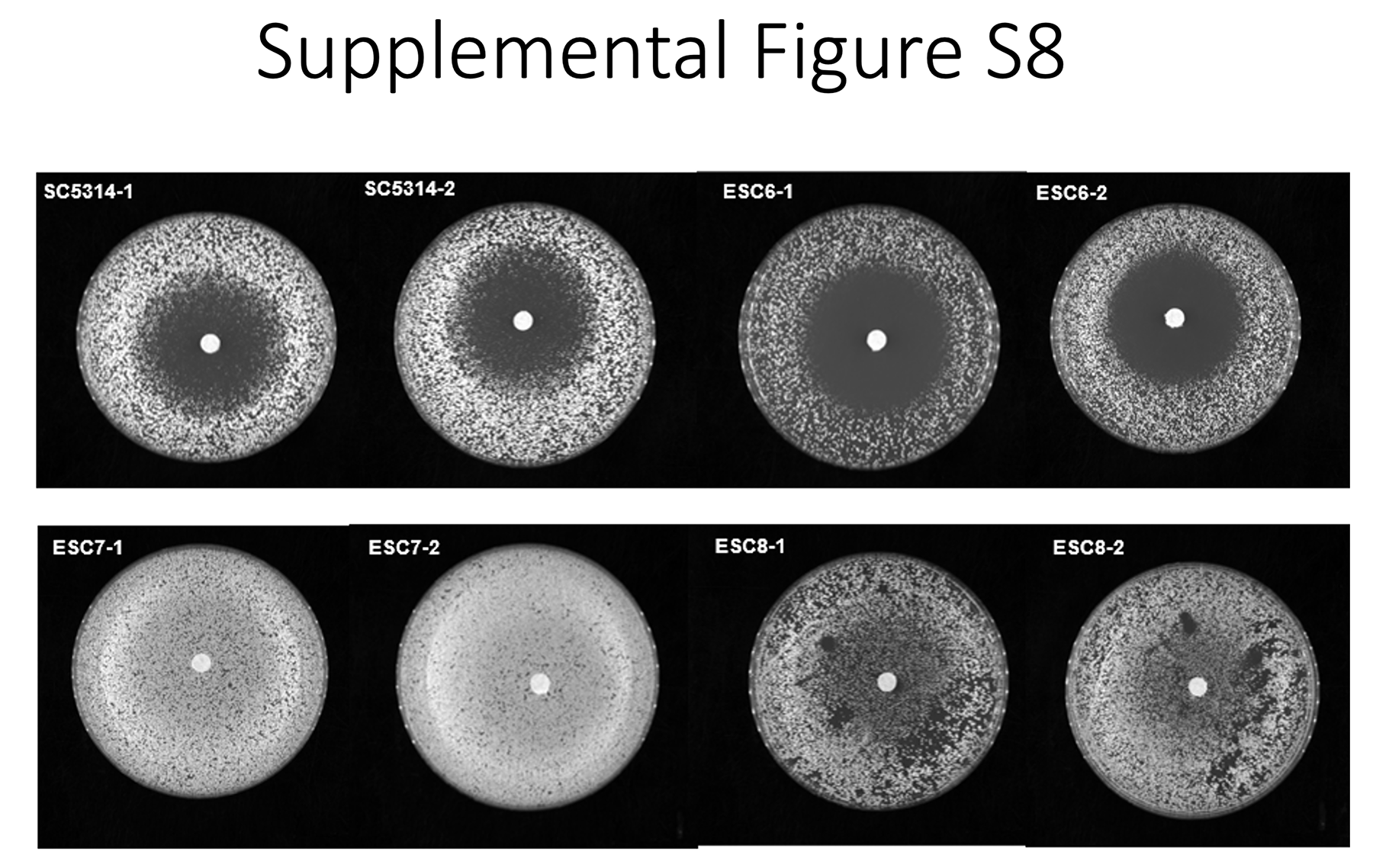

Supplement: Supplement 8 — Supplemental Figure S8: Voriconazole Disk-Diffusion Images of SC5314 and Esc Strains. SC5314 and Esc strains (−1 and −2 indicate biological replicate cultures) were grown to post exponential phase and then seeded onto YPD agar at a concentration of 105 cells per plate. A 1 ug disk of voriconazole was placed in the center of the plate. These plates were incubated at 30°C for 48 hours and then imaged with the same settings and converted to black and white images. [file media-8.tif]

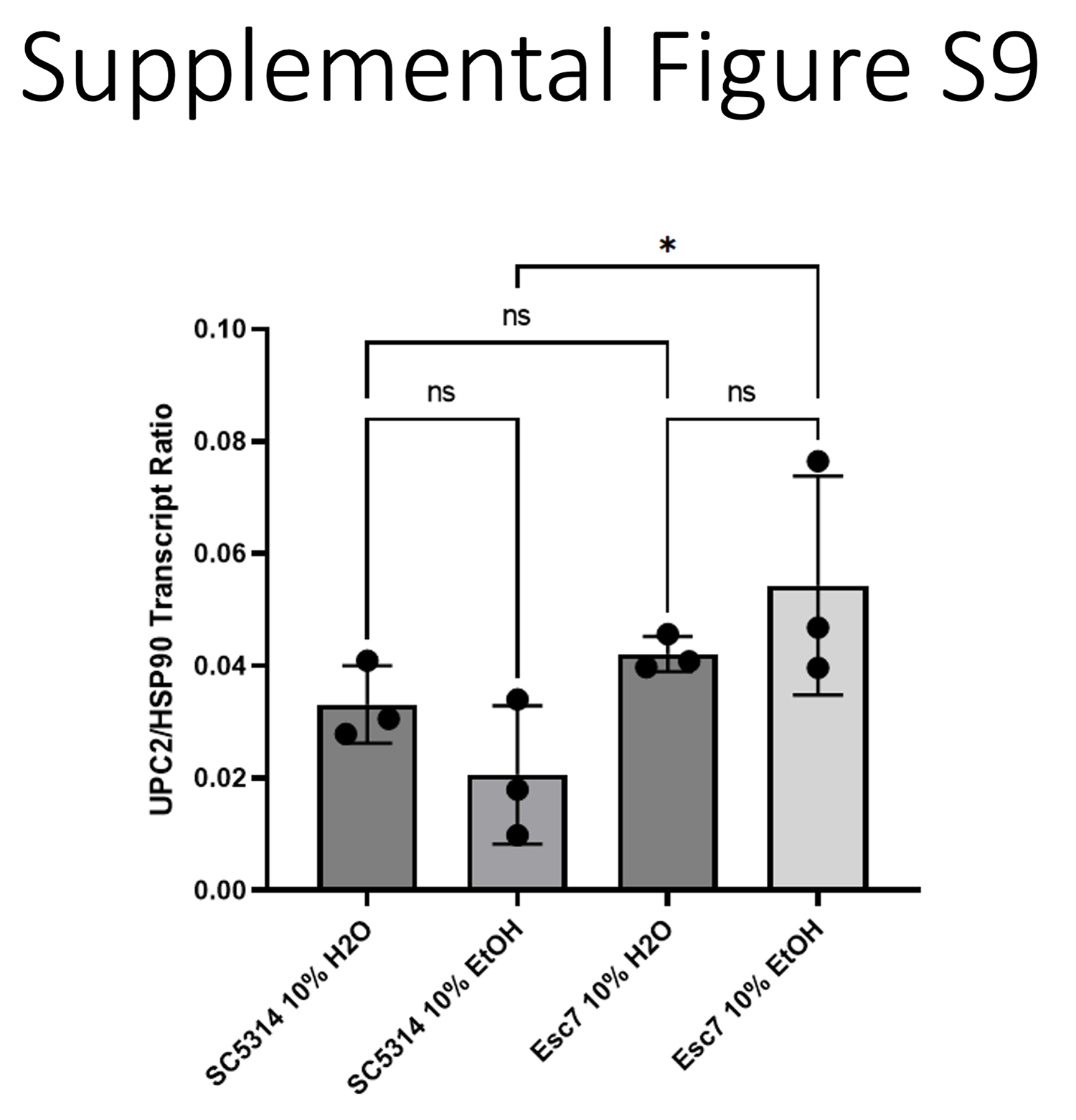

Supplement: Supplement 9 — Supplemental Figure S9: UPC2 to HSP90 Ratio of SC5314 vs Esc7 in different conditions. UPC2 to HSP90 Ratio of SC5314 vs Esc7 in different conditions. Arbitrary transcript values from RNA-seq data were used to calculate transcript ratios of UPC2 to HSP90 and were plotted (y-axis). Ordinary one-way ANOVA used for statistical significance. NS = not significant. * = p-value of 0.0377. [file media-9.tif]
