# Supplementary material for: Selection of Ethanol Tolerant Strains of Candida albicans by Repeated Ethanol Exposure Results in Strains with Reduced Susceptibility to Fluconazole
Source: bioRxiv. 2023 Nov 10:2023.09.13.557677. Originally published 2023 Sep 14. Preprint. [Version 2] doi: 10.1101/2023.09.13.557677 (PMC10515905; doi:10.1101/2023.09.13.557677)
Supplement: Supplement 23 — Supplemental Table S14: Primers used in this study. [file media-23.docx]

Supplemental Table S14 Primers used in this study

| **Primer Name For qPCR^a^** | **Sequence** | **Source** |
| --- | --- | --- |
| ERG11-FW | GCTAATTCTGTTTCATTTAACTCTTCTGAT | [1] |
| ERG11-RV | GGACCAGCTTCGGTATCCAAA |  |
| ERG2-FW | CAGCAATTGGGACTGAAGGT | [2] |
| ERG2-RV | TTCGGGAATCAATGCACCAG |  |
| ERG4-FW | CTTCGGAAGGTCAATCTTGG | [2] |
| ERG4-RV | GTCCAAACACCGGGTAAAGT |  |
| ERG5-FW | GAAGAGCAATTGCGTGTGAG | [2] |
| ERG5-RV | TGGTGGACGGTATCTCAAAG |  |
| ERG6-FW | AGATGCTGCTTCTGTTGCTG | [2] |
| ERG6-RV | GGAATGAAGAACCCCAACC |  |
| ERG25-FW | TATTTCATTGGTGGATACTCTTCATCTT | [1] |
| ERG25-RV | GGACCAGCTTCGGTATCCAAA |  |
| TPS1-FW | TCGCAAGGGTGTCTTGATCTTAT | [3] |
| TPS1-RV | AACAATCAAGGCACCATTAAGTGA |  |
| TPS2-FW | TTGCTGTTGGTCCTGCATCA | [3] |
| TPS2-RV | GGCGAGGTTCGTTCAAATGT |  |
| CDR1-FW | GGTCAACTTGTAATGGGTC | [4] |
| CDR1-RV | AGGACGATAAAGGGCATA |  |
| HSP90-FW | GGGAATCTAACGCTGGTGGTAA | [5] |
| HSP90-RV | TTCGGTTTCTGGAACTTCTTTT |  |
| ADH1-FW | CACTCACGATGGTTCATTCG | [6] |
| ADH1-RV | AAGATGGTGCGACATTGG |  |
| ADH2-FW | AAATGGTTGAACGGCTCTTG | [7] |
| ADH2-RV | GACGGTGACACCAGCACATAAG |  |
| ADH3-FW | ATTCCGACAAATACATTAAAATTAGAGG | This Study |
| ADH3-RV | AATAACCACCAAAATTGAAAATAACTT |  |
| ADH4-FW | TACTGATTCTTATGGATTATATCAAGGA | This Study |
| ADH4-RV | AAAAATCTAACAATTGGAACAATATCAG |  |
| ADH5-FW | ACCTGCAAGGGCTCATTCTG | [8] |
| ADH5-RV | CGGCTCTCAACTTCTCCATA |  |
| ALD4-FW | TTATGCCGTTGAATGTGCTC | [7] |
| ALD4-RV | CTTTGCCCGTGATTTTATCAGC |  |
| ALD5-FW | TGTTGTTACCGGTGGTGCTA | [7] |
| ALD5-RV | CAACGGCTTCGTCAACAGTA |  |
| ACS1-FW | ATTTGCCAGCTTGGTTCATC | [7] |
| ACS1-RV | CACCCTTTTTAACCCCCAAT |  |
| ACS2-FW | CTCAAGGATTTTTCGGTCCA | [7] |
| ACS2-RV | ATTCACCACCCAAAAACCAA |  |
| MDR1-FW | ACATAAATACTTTGCCCATCCAGAA | [9] |
| MDR1-RV | AAGAGTTGGTTTGTAATCGGCTAAA |  |
| SPL1-FW | AAAGGATACTGTGTTAGTTTCTATTATG | This Study |
| SPL1-RV | TATTTTCACGACATATTTTACCAATTTC |  |
| ACT1-FW | GTTGGTGATGAAGCCCAATC | [10] |
| ACT1-RV | CCCAGTTGGAAACAATACCG |  |
| CDR2-FW | GCCAATGCTGAACCGACA | [4] |
| CDR2-RV | ACCAGCCAATACCCCACA |  |

^a^Forward (FW) and reverse (RV) sequences used for primers and their corresponding gene target are listed. For primers designed for this study using NCBI primer blast, the targets were verified by Sanger sequencing.

**References**

1. Nailis H, Vandenbosch D, Deforce D, Nelis HJ, Coenye T. Transcriptional response to fluconazole and amphotericin B in Candida albicans biofilms. Res Microbiol. 2010 May;161(4):284–92.

2. Su H, Han L, Ding N, Guan P, Hu C, Huang X. Bafilomycin C1 exert antifungal effect through disturbing sterol biosynthesis in Candida albicans. J Antibiot (Tokyo). 2018 Apr;71(4):467–76.

3. Guirao-Abad JP, Sánchez-Fresneda R, Román E, Pla J, Argüelles JC, Alonso-Monge R. The MAPK Hog1 mediates the response to amphotericin B in Candida albicans. Fungal Genet Biol. 2020 Mar;136:103302.

4. Li WJ, Liu JY, Shi C, Zhao Y, Meng L ning, Wu F, et al. FLO8 deletion leads to azole resistance by upregulating CDR1 and CDR2 in Candida albicans. Res Microbiol. 2019 Sep;170(6–7):272–9.

5. Dai B, Wang Y, Li D, Xu Y, Liang R, Zhao L, et al. Hsp90 Is Involved in Apoptosis of Candida albicans by Regulating the Calcineurin-Caspase Apoptotic Pathway. Nielsen K, editor. PLoS ONE. 2012 Sep 18;7(9):e45109.

6. Bakri MM, Rich AM, Cannon RD, Holmes AR. *In vitro* expression of *Candida albicans* alcohol dehydrogenase genes involved in acetaldehyde metabolism. Mol Oral Microbiol. 2015 Feb;30(1):27–38.

7. Nieminen MT, Novak-Frazer L, Rautemaa V, Rajendran R, Sorsa T, Ramage G, et al. A Novel Antifungal Is Active against Candida albicans Biofilms and Inhibits Mutagenic Acetaldehyde Production In Vitro. Bassilana M, editor. PLoS ONE. 2014 May 27;9(5):e97864.

8. Gonçalves B, Bernardo R, Wang C, Schröder MS, Pedro NA, Butler G, et al. Effect of progesterone on Candida albicans biofilm formation under acidic conditions: A transcriptomic analysis. Int J Med Microbiol. 2020 Apr;310(3):151414.

9. Thomas E, Roman E, Claypool S, Manzoor N, Pla J, Panwar SL. Mitochondria Influence *CDR1* Efflux Pump Activity, Hog1-Mediated Oxidative Stress Pathway, Iron Homeostasis, and Ergosterol Levels in Candida albicans. Antimicrob Agents Chemother. 2013 Nov;57(11):5580–99.

10. Junier A, Weeks A, Alcaraz Y, Kumamoto CA. Bypass of Dfi1 Regulation of Candida albicans Invasive Filamentation by Iron Limitation. Mitchell AP, editor. mSphere. 2022 Feb 23;7(1):e00779-21.
