# Supplementary material for: Selection of Ethanol Tolerant Strains of Candida albicans by Repeated Ethanol Exposure Results in Strains with Reduced Susceptibility to Fluconazole
Source: bioRxiv. 2023 Sep 14:2023.09.13.557677. Preprint. [Version 1] doi: 10.1101/2023.09.13.557677 (PMC10515905; doi:10.1101/2023.09.13.557677)
Supplement: Supplementary file 1 [file NIHPP2023.09.13.557677v1-supplement-1.pdf]

bioRxiv preprint doi: https://doi.org/10.1101/2023.09.13.557677; this version posted September 14, 2023. The copyright holder for this preprint (which was not certified by peer review) is the author/funder, who has granted bioRxiv a license to display the preprint in perpetuity. It is made available under aCC-BY 4.0 International license.

Supplemental Information

Supplemental Figure S1: Quantification of small colony variants throughout passaging of four separate populations of cells that were exposed repeatedly to increasing concentrations of ethanol in medium A or repeatedly exposed to water in medium A.

Supplemental Figure S2: Enc and Wnc survival in 10% ethanol.

Supplemental Figure S3: Principal component analyses for SC5314 vs Esc6, Esc7, or Esc8 in 10% ethanol from RNA sequencing.

Supplemental Figure S4: Gene Ontology Terms of the Top 20 Processes of Significantly Enriched Transcripts in Esc strains exposed to ethanol compared to SC5314 exposed to ethanol..

Supplemental Figure S5: Histogram Fit Curve of Esc6 Allele Depth Ratio.

Supplemental Figure S6: MIC50 values from broth-microdilution experiments of SC5314 and Esc6, Esc7, and Esc8.

Supplemental Figure S7: UPC2 to HSP90 Ratio of SC5314 vs Esc7 in different conditions.

Supplemental Table S1 Transcripts showing significant changes in abundance between SC5314 with ethanol vs water medium by RNASeq

Supplemental Table S2 Transcripts showing significant changes in abundance between Esc7 with ethanol vs water medium by RNASeq

Supplemental Table S3 Transcripts showing significant changes in abundance between Esc7 with ethanol vs SC5314 with ethanol medium by RNASeq

Supplemental Table S4 Transcripts showing significant changes in abundance between Esc7 with water vs SC5314 with water medium by RNASeq

bioRxiv preprint doi: https://doi.org/10.1101/2023.09.13.557677; this version posted September 14, 2023. The copyright holder for this preprint (which was not certified by peer review) is the author/funder, who has granted bioRxiv a license to display the preprint in perpetuity. It is made available under aCC-BY 4.0 International license.

Supplemental Table S5 Transcripts showing significant changes in abundance between Esc6 with ethanol vs water medium by RNASeq

Supplemental Table S6 Transcripts showing significant changes in abundance between Esc8 with ethanol vs water medium by RNASeq

Supplemental Table S7 Transcripts showing significant changes in abundance between Esc6 with ethanol vs SC5314 with ethanol medium by RNASeq

Supplemental Table S8 Transcripts showing significant changes in abundance between Esc8 with ethanol vs SC5314 with ethanol medium by RNASeq

Supplemental Table S9 Transcripts showing significant changes in abundance between Esc6 with water vs SC5314 with water medium by RNASeq

Supplemental Table S10 Transcripts showing significant changes in abundance between Esc8 with water vs SC5314 with water medium by RNASeq

Supplemental Table S11 Esc6 Genomic Polymorphisms

Supplemental Table S12 Esc7 Genomic Polymorphisms

Supplemental Table S13 Esc8 Genomic Polymorphisms

Supplemental Table S14: Primers used in this study.
